# S100A9 protein is a novel ligand for the CD85j receptor and its interaction is implicated in the control of HIV-1 replication by NK cells

**DOI:** 10.1186/1742-4690-10-122

**Published:** 2013-10-24

**Authors:** Vincent Arnold, Jean-Saville Cummings, Uriel Y Moreno-Nieves, Céline Didier, Adrien Gilbert, Françoise Barré-Sinoussi, Daniel Scott-Algara

**Affiliations:** 1 Department of Virology, Unité de Régulation des Infections Rétrovirales, Institut Pasteur, 25 rue Dr Roux, Paris 75015, France; 2 Present address: INSERM, U897, ISPED, Université Bordeaux Segalen, Bordeaux, France; 3 Present address: Weatherall Institute of Molecular Medicine, John Radcliffe Hospital, Headington, UK, OX3 9DS, England

**Keywords:** HIV-1, NK cells, CD85j receptor, Ligand, S100A9 protein

## Abstract

**Background:**

The reportedly broad expression of CD85j across different immune cell types suggests an importance for this molecule in the human immune system. Previous reports have shown that this receptor interacts with several HLA class-I molecules, as well as with some viral proteins. We have demonstrated that the subset of CD85j + Natural Killer (NK) cells efficiently controls human immunodeficiency virus type 1 (HIV-1) replication in monocyte-derived dendritic cells (MDDC) *in vitro* and this led us to hypothesize that the CD85j + NK cell-mediated anti-HIV activity in MDDC is specifically dependent on the interaction between the CD85j receptor and unknown non-HLA class-I ligand(s).

**Results:**

In this study, we focused our efforts on the identification of these non-described ligands for CD85j. We found that the CD85j receptor interacts with a calcium-binding proteins of the S100 family; namely, S100A9. We further demonstrated that HIV-1 infection of MDDC induces a modulation of S100A9 expression on surface of the MDDC, which potentially influences the anti-HIV-1 activity of human NK cells through a mechanism involving CD85j ligation. Additionally, we showed that stimulation of NK cells with exogenous S100A9 enhances the control of HIV-1 infection in CD4+ T cells.

**Conclusions:**

Our data show that S100A9 protein, through ligation with CD85j, can stimulate the anti-HIV-1 activity of NK cells.

## Background

Human leukocyte immunoglobulin-like receptors (LILRs), also referred as immunoglobulin-like transcripts (ILTs) and leukocyte immunoglobulin-like receptors (LIRs), are a family of innate immune receptors that recognise self-antigens [1]. The LILRs molecular family consists of at least 10 genes coding for proteins of the Immunoglobulin superfamily. Some products of these genes, such as CD85j (alternatively: LILRB1, ILT2, or LIR-1), are surface membrane inhibitory receptors which reduce cellular activation after encountering their cognate ligands [2]. CD85j is found on 23–77% of human Natural Killer (NK) cells, on a small percentage of T lymphocytes (4–20%), on most B cells, monocytes and dendritic cells (DC) [3-5]. This broad expression of the inhibitory CD85j across the immune system suggests an importance for this molecule in the control of immune activation in humans. The data of the literature indicate that CD85j expression is associated with diseases both where cellular immune responses are ineffective, such as cytomegalovirus (CMV) infection [6], and also where an over-active cellular immune response is detrimental to the host [7,8].

The extracellular region of the CD85j receptor interacts with several classical and non-classical HLA class-I molecules, as well as with the viral protein UL18 from human CMV (HCMV) [4]. It is the only reported member of the LILR family that recognizes a non-self antigen. When CD85j is engaged by specific cross-linking antibodies (Abs) or by HLA class-I molecules, it delivers inhibitory signals to dendritic cells, NK cells and T lymphocytes [3,5,9-12]. Escape mechanisms involving CD85j triggering have been reported, for instance, expression of CD85j ligands such as HLA-G on tumor cells, has been suggested to be an escape mechanism of gamma delta T-cell antitumoral activity [13] or continuous ligation of CD85j on DC can induce suppression of T cell responses[14]. Also it has been reported that the CD85j–UL18 interaction is a mechanism of viral escape from the NK cell-mediated lysis [15,16]. However, other groups have described it as a mechanism that leads to the killing of HCMV-infected cells by NK cells and T lymphocytes via non-HLA-restricted targeting [17-19]. Despite our incomplete understanding of the CD85j-UL18 inhibitory or activating mechanisms, this interaction appears to be of central importance in the immune response to HCMV infection. We have previously shown that the CD85j + NK cell subset control more efficiently HIV-1 replication in monocyte-derived dendritic cells (MDDC) compared to their CD85j- NK cell counterpart. This CD85j + NK cell mediated anti-HIV-1 activity is dependent on cell-to-cell contact, and on the interaction between the CD85j receptor and non-HLA class-I ligand(s) expressed on MDDC [20]. In the present study, we focused our efforts on the identification of unknown non-HLA class-I ligands for CD85j. We observed that S100A9, a calcium-binding protein of the S100 family, is expressed by MDDC in response to HIV-1 infection and is potentially implicated in the anti-HIV-1 activity of human NK cells through the CD85j ligation and signalisation.

## Results

### Identification of non-HLA class-I ligand(s) for the CD85j receptor

**Figure 1:**
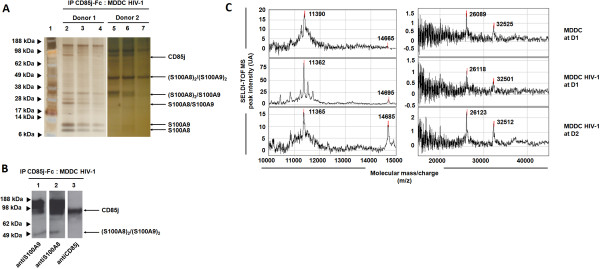
**Identification of CD85j ligand(s) expressed by HIV-1-infected MDDC. (A)** MDDC were lysed at day 2 post HIV-1 infection. After blocking CD85j receptors and HLA class-I ligands as described in Materials and Methods, CD85j-Fc fusion protein was used to capture and purify proteins from uninfected or HIV-1-infected MDDC lysates onto the Protein chip array. Eluted proteins from the column were separated by electrophoresis in non-reducing conditions and revealed by silver staining. Lane 1, Seeblue® Plus2 Pre-Stained Standard, lanes 2–7, subsequent eluted fractions of HIV-1-infected MDDC lysates after pre-incubation with the CD85j-Fc-coupled gel from two representative donors (lanes 2–4, donor 1; lanes 5–7, donor 2). The proteins were identified as belonging to S100A8/A9 complex (S100A8, S100A9 and S100A8/A9) by protein sequencing. **(B)** Eluted fractions of HIV-1-infected MDDC lysates were analyzed by immunoblotting using anti-S100A9 (lane 1), anti-S100A8 (lane 2) and anti-CD85j (lane 3) mAbs. **(C)** CD85j-Fc fusion protein was used to capture and purify proteins from uninfected or HIV-1-infected MDDC lysates, where HLA-I molecules were blocked, onto the Protein chip Array.

We used Protein chip array technology to initially identify novel CD85j ligands expressed by uninfected or HIV-1-infected MDDC. To isolate non-HLA class-I ligand(s), we performed an immunoprecipitation procedure with recombinant CD85j-Fc proteins targeting cognate ligands, different from HLA class-I molecules, from uninfected or HIV-infected MDDC lysates. Thereafter, we performed an electrophoresis in non-reducing conditions followed by a silver staining (Figure 1A, two representative protein profiles) and an immunoblot (Figure 1B) to visualize the immunoprecipitated proteins. Among the different donors tested there were differences in the expression of CD85j cognate partners, for instance we observed 5 (Figure 1A, Donor 1) and 2 proteins (Figure 1A, Donor 2) in the eluted fractions of HIV-1-infected MDDC lysates. By sequence comparison we were able to identify 4 molecular forms of two proteins: S100A8 and S100A9 (Additional file 1: Figure S1). We observed S100A8 (11 kDa) and S100A9 (14 kDa) monomers, S100A8/S100A9 heterodimer (26 kDa), (S100A8)_2_/(S100A9) trimer (32 kDa), as well as (S100A8/S100A9)_2_ tetramer (49 kDa) in the eluted fractions of HIV-1-infected MDDC lysates (Figure 1A). The fractions were analyzed by immunoblotting using anti-S100A9 (lane 1), anti-S100A8 (lane 2) and anti-CD85j (lane 3) mAbs (Figure 1B). We observed a co-localization of the bands for S100A8 and S100A9 (lane 1 and 2, respectively) with CD85j (lane 3), therefore the higher molecular weight band (98 kDa) may correspond to this complex.

The interaction between CD85j receptor and eluted S100A8/A9 proteins was then verified by Protein chip array (Figure 1C). The peaks of the S100A8 monomer (11 kDa), S100A8/S100A9 heterodimer (26 kDa) and trimer (32 kDa) were consistently observed in the eluted fractions of uninfected and HIV-1-infected MDDC lysates. By contrast, the peak of S100A9 monomer protein (14 kDa) was only present in eluted fractions of HIV-1-infected MDDC lysates (Figure 1C) and not in mock-infected conditions (data not shown). These results supported those obtained by electrophoresis (Figure 1A) and the immunoblot analysis (Figure 1B).

### Analysis of CD85j/S100A8 and CD85j/S100A9 interactions

**Figure 2:**
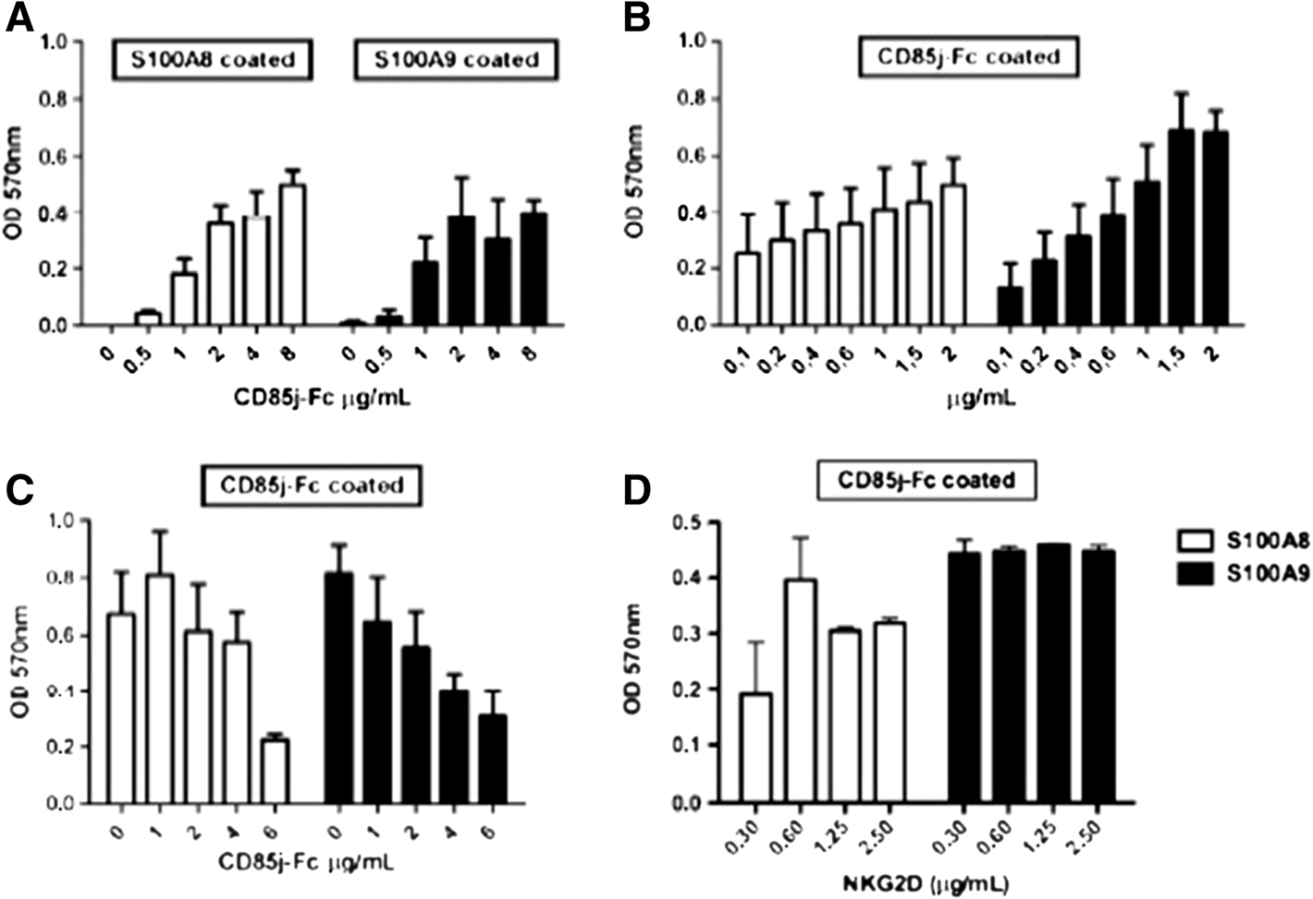
**ELISA-based CD85j/S100A8 and CD85j/S100A9 binding assay. (A)** Anti-GST mAbs well-coated microtiter plates were incubated with 4 μg/mL of recombinant GST-tagged human S100A8 or S100A9 proteins followed by incubation with increasing amounts of CD85j-Fc protein. **(B)** CD85j-Fc protein well-coated microtiter plates were incubated with increasing concentrations of recombinant GST-tagged human S100A8 or S100A9 proteins. **(C)** CD85j-Fc protein well-coated were incubated with 4 μg/mL of human S100A8 or S100A9 proteins in the presence of increasing amounts of soluble CD85j-Fc protein. **(D)** CD85j-Fc protein well-coated were incubated with 4 μg/mL of human S100A8 or S100A9 proteins in the presence of increasing concentrations of soluble NKG2D-Fc protein. Each point is the mean ± SE of 4 determinations.

The CD85j/S100A9 interaction was then analysed in comparison to CD85j/S100A8 by ELISA test (Figure 2). The CD85j-Fc protein bound to both recombinant S100A8 and S100A9 monomer proteins in a dose-dependent manner (Figure 2A). We also conducted the reverse experiment where S100A8 or S100A9 proteins at different concentrations were titrated over immobilized CD85j-Fc (Figure 2B); similarly, we observed a dose-dependent interaction between the monomeric proteins S100A8 and S100A9, and the CD85j-Fc protein, however a better binding of S100A9 monomer to CD85j-Fc was found. To determine the specificity of the S100A8/CD85j and S100A9/CD85j interactions, we blocked the binding using increasing concentration of recombinant CD85j-Fc protein (from 1 to 6 μg/mL). As shown in Figure 2C, increased concentrations of soluble CD85j-Fc protein were able to clearly block the interaction of both S100A9 protein and in a lesser extend S100A8 protein with immobilized-CD85j-Fc. We also performed control blocking experiments using NKG2D-Fc protein (as a non-related control fusion protein) at different concentration (ranging from 0.3 to 2.5 μg/mL). We did not observe any block of S100A8/CD85j and S100A9/CD85j interactions regardless the NKG2D concentration used (Figure 2D), which confirms the specificity of these interactions. We then compared the interaction of S100A9 monomers and homotetramers with the CD85j fusion protein. A higher binding capacity of S100A9 tetramers was detected when compared to monomers (Additional file 2: Figure S2).

### Modulation of S100A8, S100A9 and S100A8/A9 complex on the surface of MDDC in response to a productive HIV-1 infection

**Figure 3:**
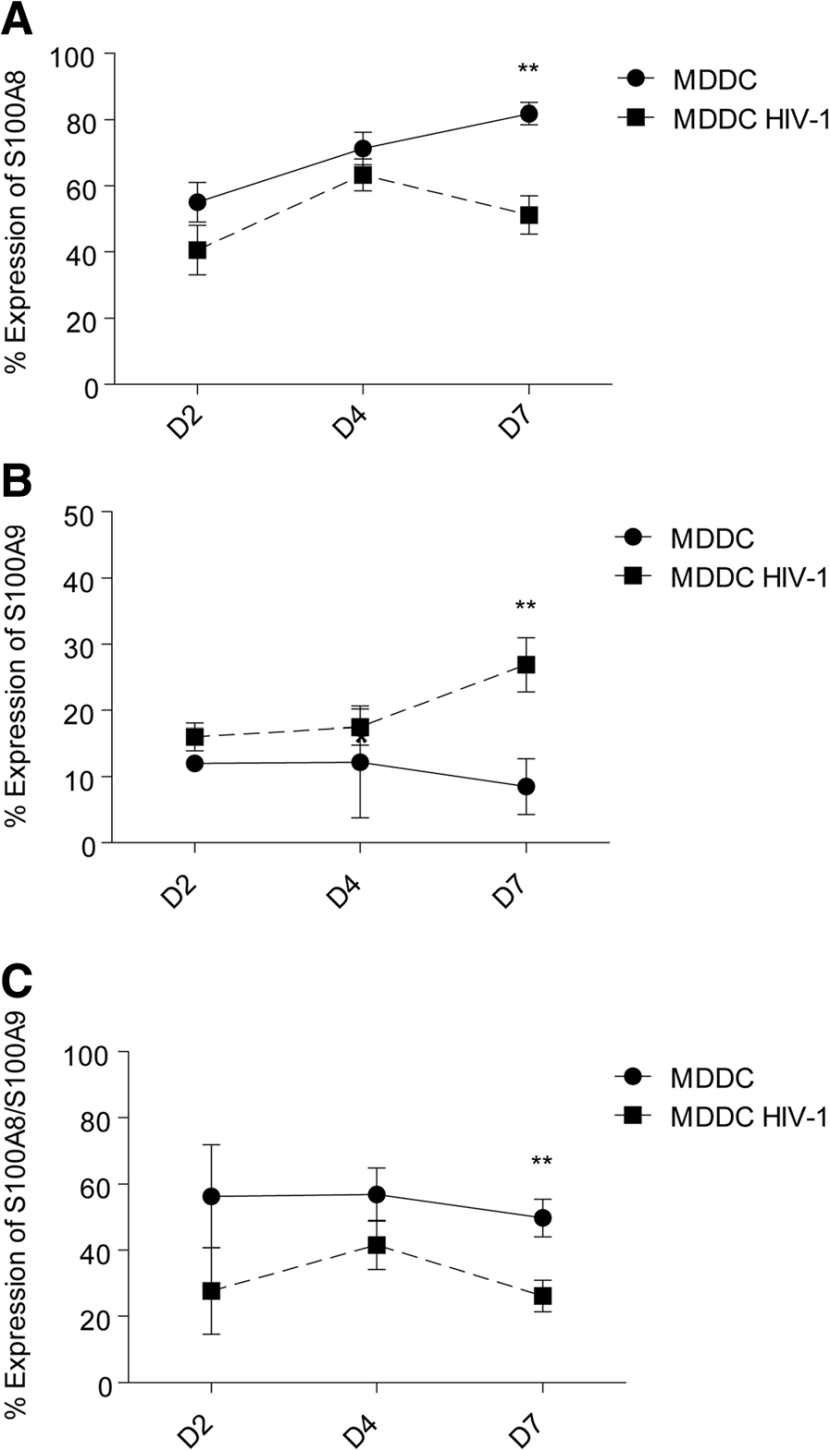
**Surface expression of S100A8, S100A9, and S100A8/S100A9 proteins on MDDC in response to HIV-1.** Kinetics of the expression of S100A8 **(A)**, S100A9 **(B)** and S100A8/S100A9 complex **(C)** at the surface of MDDC (solid black line) or HIV-1-infected MDDC (dotted black line). Results of 10 independent experiments are summarized and expressed as mean ± SE of percentage of MDDC expressing S100A8, S100A9, or S100A8/S100A9. **p < 0.01.

Our previous results suggested that CD85j ligands distinct from HLA class-I molecules are preferentially expressed on HIV-1-infected MDDC [20]. To explore the possibility that the expression pattern of S100A8/A9 proteins is modulated at the surface of MDDC in response to HIV-1 infection, we monitored their expression by flow cytometry over time (Figure 3). The surface expression of S100A9 was increased on MDDC in response to HIV-1 infection (Figure 3B), whereas surface expression levels of S100A8 and S100A8/S100A9 were both decreased (Figure 3A and 3C). Further analysis of the expression of S100 proteins following HIV-1 infection showed that S100A9 expression was increased and S100A8/S100A9 complex expression was decreased in MDDC that do not replicate the virus, whereas almost none of these proteins were expressed by MDDC replicating the virus (data not shown). Additionally, the HIV-1-induced decrease of S100A8 expression was observed on both MDDC replicating or not the virus (data not shown). Moreover, we were able to show that these modulations in the expression of S100A8/A9 proteins are HIV-1 dose-dependant (Additional file 3: Figure S3). These results demonstrate distinct modulations of S100A8, S100A9 and S100A8/S100A9 proteins on MDDC in response to a productive HIV-1 infection *in vitro*.

### S100A9 tetramer stimulation modulates NK cell functions

**Figure 4:**
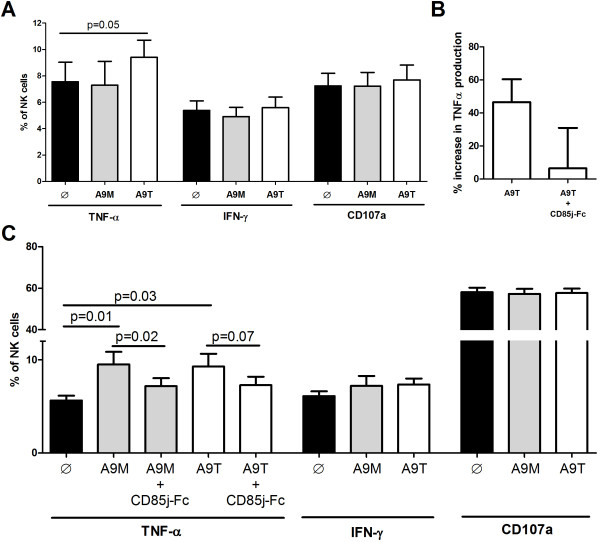
**Cytokine production and CD107a expression on NK cell after stimulation by S100A9 monomer and tetramer proteins. (A)** Intracellular expression of TNF-α and IFN-γ and surface expression of CD107a on NK cells unstimulated, stimulated by S100A9 monomers or tetramers for 4 hours at 37°C (black, grey and white bars respectively). Graph shows cumulative results from 7 independent experiments. **(B)** NK cells were stimulated with S100A9 tetrater or with an equimolar combination of CD85j-Fc + S100A9 tetramer for 4 h at 37°C. For the later stimulation, CD85j-Fc and S100A9 tetramer were co-incubated for 2 hours at 37°C prior stimulation of NK cells. Graph represents the percentage of increase in TNF-α expression following stimulation compared to the unstimulated NK cells. Graph shows cumulative results from 4 independent experiments. **(C)** Expression of intracellular TNF-α and IFN-γ and surface CD107a by NK cells unstimulated, pre-stimulated by S100A9 monomer or tetramers (black, grey and white bars respectively) following a secondary stimulation by K562 target cells for 4 h at 37°C. For the blocking experiment regarding intracellular TNF-α expression, NK cells were stimulated with an equimolar combination of CD85j-Fc + S100A9 tetramer. Graph shows cumulative results from 8 independent experiments. Results are expressed as mean ± SE and *p* values are indicated.

Following the experiments showing the interaction between CD85j and S100A9, we wanted to examine whether or not exogenous stimulation by these proteins can influence NK cell responses (Figure 4). As it is known that tetramers of proteins in some cases can have greater biological activity than their monomeric counterparts [21], we studied the effect of the monomeric and homotetrameric forms of S100A9 on the modulation of NK cell responses. First, we tested the modulation of NK cell function after stimulation of purified NK cells by S100A9 proteins. As shown in Figure 4A, a clear increase of TNF-α production was observed when NK cells were stimulated with S100A9 tetramer whereas no modulation of TNF-α production was detected following S100A9 monomer stimulation; stimulation of NK cells with either the monomeric or the tetrameric form of S100A9 had no impact on the production of IFN-γ or degranulation. Additionally, we observed that the increase in TNF-α production following stimulation of NK cells by S100A9 tetramers can be reduced by the co-incubation of S100A9 tetramers with soluble CD85j-Fc prior stimulation of NK cells (Figure 4B). We next evaluated the ability of S100A9 monomer- and tetramer-stimulated NK cells to recognize the HLA–deficient target K562 cell line (Figures 4C). As expected, stimulation of resting NK cells with K562 cells induced cytokine secretion and an increase of CD107a surface expression (data not shown). Pre-stimulation of NK cells by S100A9 monomers and tetramers (Figure 4C) induced a significant increase in TNF-α production but had no significant effect in the production of IFN-γ and the expression of CD107a. The increased TNF-α production can be abrogated by stimulation of NK cells with S100A9 tetramers combined with CD85j-Fc (Additional file 4: Figure S4). Importantly, stimulation of NK cells with S100A8 alone or followed by K562 target recognition did not modulate the production of cytokines nor the degranulation (data not shown). These results therefore suggest that S100A8 monomers stimulation does not induce a modulation of NK cell responses, whereas stimulation of NK cells by S100A9 (either its monomeric or tetrameric form) modulates the production of TNF-α.

### S100A9 tetramer stimulation potentiates anti-HIV-1-mediated NK cell activity

**Figure 5:**
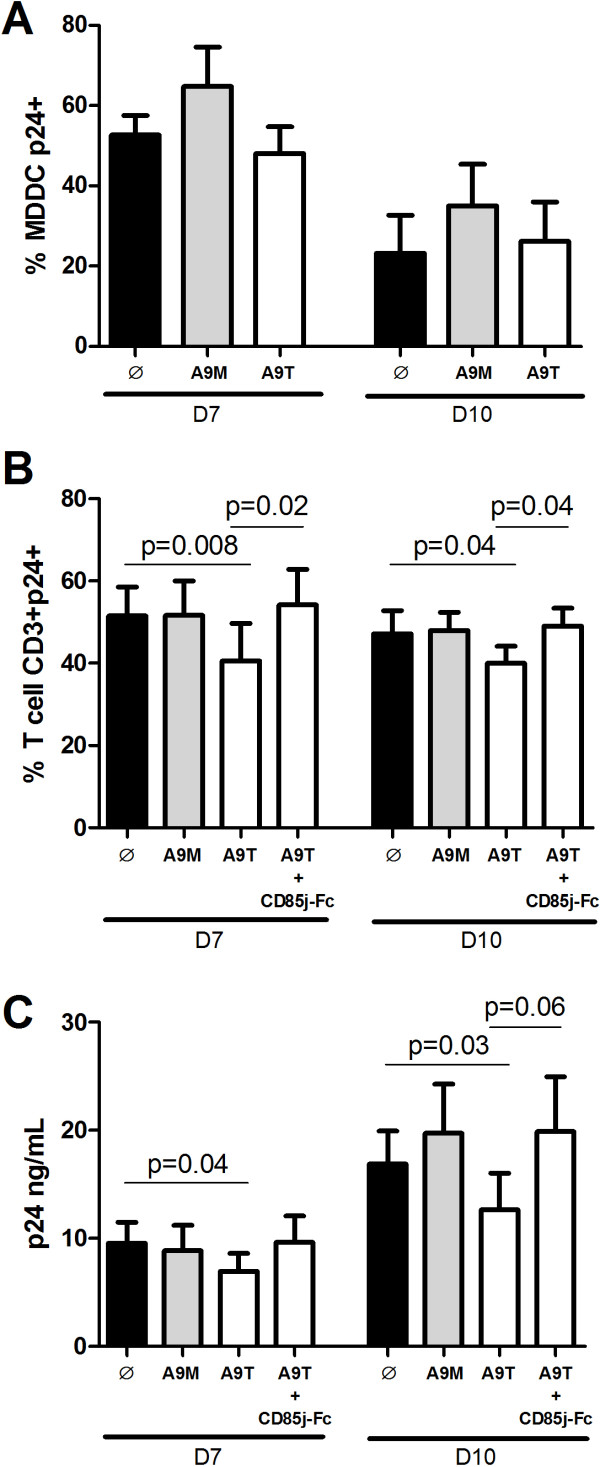
**Anti-HIV-1 activity of NK cell stimulated by S100A9 monomer or tetramer proteins.** MDDC or CD4+ T cells were infected by HIV-1 and then cultured with unstimulated, S100A9 monomer- or S100A9 tetramer-stimulated NK cells (black, grey and white bars respectively) at the ratio E/T of 1:5. **(A-B)** HIV-1 replication was measured by intracellular p24 expression in MDDC and CD4+ T cells after 7 and 10 days of culture. **(A)** Graph represents cumulative results from 4 independent experiments showing the percentage of p24+ MDDC. **(B)** Graph represents cumulative results from 8 independent experiments showing the percentage of p24+ CD4+ T cells. **(C)** Graph represents cumulative results from 6 independent experiments showing the amount of p24 HIV-1 antigen in the supernatant of HIV-1-infected CD4+ T cells in culture with unstimulated, S100A9 monomer- or S100A9 tetramer-stimulated NK cells (black, grey and white bars respectively). For blocking experiments, NK cells were stimulated with an equimolar combination of CD85j-Fc + S100A9 tetramer. Results are expressed as mean ± SE and *p* values are shown.

We have previously shown that the engagement of the NK CD85j receptor with non-HLA class-I ligand(s) plays a critical role in the anti-HIV-1-mediated NK cell activity on MDDC [20]. Since S100A9 proteins modulate NK cell activity (Figure 4), we then tested the capacity of S100A9 stimulated NK cells to control viral replication (Figure 5). Viral replication, measured by GFP expression, was decreased in MDDC after 7 days of culture with NK cells compared to MDDC cultured without NK cells (12.5% vs 27.9% HIV/GFP + MDDC, respectively, data not shown). However, S100A9 monomer- or tetramer-stimulated NK cells do not have a better capacity to control HIV-1 replication in MDDC than unstimulated NK cells (Figure 5A) instead, S100A9 monomer stimulated-NK cells seem to slightly increase HIV-1 viral replication.

We also tested the impact of the interaction CD85j/S100A9 in the NK cell-mediated control of HIV-1 infection in purified autologous CD4+ T cells by pre-stimulating NK cells with exogenous S100A9 proteins. Of note, S100A8 and S100A9 proteins are constitutively expressed by phagocytic myeloid cells but they are not expressed or induced in CD4+ T cells [22]. As HIV-1 infection induces a down regulation of CD4 expression, we studied the p24 expression in CD3+ T cells as a measure of viral replication; cells were cultured at a NK/CD4 T cell ratio of 1:5, which is close to physiological conditions, without any NK cell cytokine activation. Pre-stimulation of NK cells by S100A9 tetramers induced a significant decrease in the percentage of CD3+ T cells replicating the virus (Figure 5B), at day 7 and 10 post-infection, and this decrease was abrogated when NK cells were pre-stimulated with a combination of S100A9 tetramers and CD85j-Fc. Additionally, measure of p24 in the supernatant of the co-culture showed a significant decrease in the viral production by CD4+ T cells following their culture with S100A9 tetramer-stimulated NK cells (Figure 5C), and this observed decrease in the amount of p24 antigen trended to be abrogated when NK cells were pre-incubated with a combination of S100A9 tetramers and CD85j-Fc. Pre-stimulation of NK cells by S100A9 monomers induced no change in viral replication (Figure 5B and 5C); neither S100A8 pre-stimulation had an effect in the control of HIV-1 infection (data not shown). Taken together, these results show that S100A9 tetramer-stimulation of NK cells enhances the NK cell-mediated anti-HIV-1 responses.

## Discussion

By using different techniques (co-immunoprecipitation, electrophoresis, ELISA, and Protein chip array technology), we were able to isolate and identify new MDDC-expressed ligands for the CD85j receptor, namely S100A8 and S100A9 proteins (Figures 1 and 2). We demonstrated that *in vitro* HIV-1 infection of MDDC induces a modulation of S100A8, S100A9 and S100A8/S100A9 surface expression (Figure 3). Overall, our data indicate that S100A9 (but not S100A8) may influence the anti-HIV-1 activity of human NK cells through CD85j engagement.

We showed that only S100A9 seems to directly regulate cytokine production (TNF-α) by NK cells and after secondary activation by target cells (K562) through CD85j engagement (Figure 4). To our knowledge, it has never been described that the engagement of the inhibitory receptor CD85j on peripheral NK cells could enhance cytokine secretion. Li C. *et al. *[23] reported an increased production of pro-inflammatory cytokines including TNF-α by decidual NK cells after cross-linking of CD85j by monoclonal antibodies or stimulation with HLA-G homodimer, but they did not observe these cytokine production by peripheral NK cells after stimulation. Of note, an Immunoreceptor Tyrosine-based Switch Motif (ITSM) in the CD85j and SAP and/or EAT adaptors molecules may be implicated in the activating signal observed [23]. SAP and EAT adaptors molecules are expressed in human NK cells and induce activating signals. This finding could explain our results, by the fact that interaction of CD85j and S100A9 proteins may induce an activating signalling mediated by the ITSM motif. It is known that small changes in the ligands for inhibitory receptors represent a potent mechanism for the rapid removal of dominant inhibitory signals by changing the interaction affinity, resulting in NK cell activation [24]. Therefore, we can hypothesize that the specificity and the affinity of CD85j engagement with its ligand could influence the integration of signals and determine the threshold of cellular activation. However, we cannot rule out the possibility that S100A9 also may interact with other receptors than CD85j. The known receptors for S100A8 and S100A9 include heparin sulphate, TLR4, carboxylated N-glycans and the Receptor for Advanced Glycation End-products (RAGE) [22,25,26].

Our previous work suggested that NK cells use CD85j ligand(s) other than HLA class-I molecules to inhibit HIV-1 replication in MDDC through a cytotoxic-independent and cell-to-cell contact-dependent mechanism [20]. Here, S100A9 tetramer-stimulated NK cells appeared to have a better capacity to control HIV-1 infection than unstimulated NK cells. Therefore, we speculate that NK cells stimulated by S100A9 protein through CD85j receptor might secrete factors at the immunological synapses with target cells which have the capacity to suppress HIV-1 replication. The biological functions of S100A9 proteins can be modified by their conformational variability and multimerization. It is then possible that monomeric and tetrameric forms of S100A9 have different abilities to signal in NK cells through CD85j modulating the NK cell response. We found that S100A9 homotetramer protein interacts stronger with CD85j-Fc protein than S100A9 monomer protein (Additional file 2: Figure S2), this stronger affinity may account for the higher NK cell mediated anti-HIV-1 response observed. As S100A9 tetramers do not induce higher activation of NK cells towards K562 target cells, whereas they increase anti-HIV-1 activity, it is tempting to speculate that the signalling they induce in NK cells prompts the cells to better respond to an HIV-1 infected cell compared to a tumor cell line. Stimulation of NK cells with S100A9 proteins alone or followed by tumoral target cell induces TNF-α production. However, TNF-α has been linked to the immune activation and the enhancement of HIV-1 infection in CD4+ T cells. In this way, TNF-α secretion might not be the main way for NK cells to control HIV-1 infection, instead other mechanisms may account for the viral control such as the modification of the NK cell receptor repertoire.

Previous reports have suggested that S100A9 is involved in inflammation. Indeed, the first phagocytes, which infiltrate and dominate acute inflammatory lesions, have been shown to express S100A9 proteins[27-29]. In parallel, NK cells are recruited from the blood to the site of inflammation. Thus, the interactions between CD85j and S100A9 proteins are likely to occur during the early phase of HIV-1 infection characterised by excessive inflammatory responses through NK/DC crosstalk. This may activate CD85j-expressing NK cells (regarding their secretory function) to control HIV-1 replication in CD4+ T cells.

S100A9 and S100A8/S100A9 complexes were almost exclusively expressed by p24- MDDC and were up regulated and down regulated respectively on p24- MDDC during the course of a productive HIV-1 infection (Figure 3, Additional file 5: Figure S5 and data not shown). Overall, a productive HIV-1 infection of MDDC was needed to induce changes in the surface expressions of S100A8, S100A9 and S100A8/S100A9 on p24-MDDC. Bystander effects of a productive HIV-1 infection could explain the observed modulation and may include impairment or enhancement of soluble factors secreted by HIV-1 infected cells. Though neither Nef nor Vpu proteins, both of which are able to down regulate HLA class-I molecules [30,31], seemed to be implicated in these S100A8 and S100A9 expression modulations on MDDC in response to HIV-1 infection (Additional file 5: Figure S5 and data not shown), however, we cannot rule out an implication of other HIV-1 proteins. It remains to be determined whether these HIV-1-induced modulations on MDDC represent an established host defences against HIV-1 infection.

CD85j is highly expressed on other innate cells, such as monocytes and dendritic cells, and also on B cells, therefore it is possible that CD85j/S100A9 interaction plays a role in the modulation of the activity of those cells during HIV-1 infection. Finally, our results could have more wide implications in the response against pathogens or cancer cells. In cancer patients, accumulation of myeloid-derived suppressor cells (MDSC), which are known to impair anti-tumour immunity (notably by decreasing cytotoxic function of NK cells), are regulated by S100A9 protein [32]. According to results presented herein, the mechanism described in cancer models could be CD85j-mediated.

## Conclusion

Our results demonstrate that S100A9 protein, through ligation with CD85j, can stimulate the anti-HIV-1 activity of NK cells. This S100A9/CD85j interaction probably plays an important role in the NK/DC crosstalk and therefore could impact the establishment and the regulation of the specific antiviral/antitumor immune response.

## Methods

### Ethics statement

Blood was obtained from healthy donors through the Etablissement Français du Sang. Written informed consent was provided by study participants and/or their legal guardians according to French ethical laws. This study was approved by the Committee of Clinical Research (number 2007–23) and the Institutional Review Board (IRB, 06966; agreement from the Office for Human Research Protection / United States Department of Health and Human Services) from Institut Pasteur.

### Isolation of monocytes, NK and CD4+ T cells

Peripheral blood mononuclear cells (PBMC) were obtained after Ficoll gradient separation. Positive selection for CD14+ PBMC was performed using anti-CD14 microbeads, MS+/RS + columns and a MiniMACS separator (Miltenyi Biotec). MDDC were generated from monocytes as previously described [20]. Similarly, positive selection for CD56+ and CD4 + CD14- cells were performed using anti-CD56 and anti-CD4 microbeads respectively (Miltenyi Biotec). CD56+ and CD4 + CD14- cells were frozen in 90% fetal calf serum, 10% DMSO (Sigma). After thawing, a negative selection for CD3-CD56+ was performed using anti-CD3 microbeads (Invitrogen). The percentage of CD3-CD56+ NK cells and CD4+ cells in the isolated population, evaluated by flow cytometry, was 95%. Purification of CD85j NK cell subsets was performed as previously described [20].

### HIV-1 infection of MDDC

Immature MDDC were infected with R5 strains AD8 WT (HIV-1), HIV-1 molecular clones lacking the Nef (HIV-1ΔVpu) or Vpu (HIV-1ΔVpu) (a gift from Dr Olivier Schwartz lab, Paris, France) or with a recombinant GFP HIV-1 virus (HIV-1/GFP). We used also a single-round an HIV-1 pseudotyped virus with vesicular stomatitis virus G protein (HIV-1ΔEnv VSV). Infections were performed with each virus at a multiplicity of infection (moi) of 10^-1^ by spinoculation. MDDC were then extensively washed and seeded into plate wells.

### Lysis of MDDC and co-immunoprecipitation

HIV-1-infected or uninfected MDDC were incubated or not with 10 μg/ml of mouse anti-HLA-class I W6/32, anti-HLA-class G, anti-HLA-class E (Serotec), and anti-CD85j mAbs (Beckman Coulter), followed with donkey anti-mouse secondary reagent (Jackson Immunoresearch). All incubations with MDDC were performed for 30 min, at room temperature at day 1 or day 2 post HIV-1 infection. MDDC were harvested, centrifuged, and incubated for 40 min at 4°C in 0.5% Triton X-100 (CalBiochem) lysis buffer with protease inhibitor (Complete, Roche). After a microcentrifugation for 15 min at 10000 g, the supernatant were collected. Recombinant CD85j-Fc fusion protein (R&D System) was covalently immobilized on an amine-reactive gel column, as described by the manufacturer (ProFound Co-imunoprecipitation kit, Thermo Scientific Pierce). Subsequently, MDDC cell lysates were loaded onto the column. After extensive washing, CD85j-Fc-bound proteins were eluted from the column using the elution buffer and analyzed by protein chip array, 4-12% NuPAGE revealed by SilverQuest SilverStaining kit (Invitrogen) or by Western Blot.

### Protein chip array

The profiles of uninfected and HIV-1-infected MDDC lysates, as well as consecutively eluted fractions, were examined by surface-enhanced laser desorption/ionization time-of-flight mass spectrometry (SELDI-TOF-MS) Protein chip arrays (Ciphergen Biosystems) according to the manufacturer’s recommendations. Briefly, a PS10 protein chip was incubated with CD85j-Fc proteins at 500 μg/ml in PBS (Gibco) at 4°C for 18 h. Residual sites were blocked by washing the protein chip with 1 M ethanolamine, pH 8.0, for 30 min followed by three washes of 10 min in PBS containing 0.5% Triton X-100 (CalBiochem). Then, samples were applied on the chip. After incubation for 2 h at room temperature, unbound proteins to CD85j-Fc were removed by three successive washes of 5 min each with a buffer containing 1 mM NaCl and 5 mM HEPES (N-2-hydroxyethylpiperazine-N’-2-ethanesulfonic acid). Chip-captured proteins were air-dried and covered with a matrix (3,5-dimethoxy-4-hydroxycinnapynic acid in 50% acetonitrile and 0.5% trifluoroacetic acid), used as an absorbent for laser energy. The ionized and desorbed proteins were detected and their molecular masses displayed on the proteogram as peaks. SELDI-TOF-MS analysis was done with the Protein-Chip Biology System II software (PBS II) and Ciphergen Peaks software.

### Western blot analysis

The eluted proteins were analyzed by 4-12% NuPAGE and transferred to Immobilon-P membranes (Millipore). After blocking with 5% skimmed milk, the membrane were incubated with mouse anti-CD85j (Beckman Coulter), anti-human S100A8 (2H2) or S100A9 (1C10) (Tebu-bio) mAbs followed by secondary horseradish peroxidase-goat anti-mouse F(ab)_2_ Abs (R&D System). The proteins were revealed on Hyperfilms (Amersham) by using the ECL chemiluminescent substrate (GE Healthcare).

### Ligand binding ELISA assay

Microtiter plates (96-well) were coated with 250 ng of indicated mAbs in 0.1 M sodium carbonate buffer, pH = 9.6 overnight at 4°C. Plates were washed and blocked with 3% of bovine serum albumin in PBS (Gibco) for 2 h at 37°C. The wells were then incubated with the indicated proteins for 2 h at 37°C. Binding of CD85j-Fc fusion protein (R&D System) to immobilized GST-tagged human S100A8 or S100A9 monomer proteins (Tebu-bio) was detected by adding horseradish peroxydase-antihuman-Fc mAbs (Jackson Immunoresearch). Binding of either S100A8 monomer, S100A9 monomer or S100A9 tetramer (Proteinia, SA) to immobilized CD85j-Fc protein was detected by adding horseradish peroxydase-conjugated goat anti-GST polyclonal antibody (GE Healthcare). For blocking experiments, S100A8 or S100A9 proteins were added to the CD85j-Fc-immobilized wells in the presence of varying amounts of soluble CD85j-Fc or NKG2D-Fc (R&D System) protein. After a final wash step, a substrate reagent containing tetramethylbenzidine and hydrogen peroxide was added. Colours absorbance was measured at 570 nm using a spectrophotometer.

### Analysis of S100A8, S100A9 and S100A8/S100A9 surface expressions

Uninfected or HIV-1-infected MDDC were stained with either mouse anti-human S100A8 (2H2) or S100A9 (1C10) mAbs followed with PE-conjugated donkey anti-mouse secondary reagent (Jackson Immunoresearch). MDDC were directly stained with PE-conjugated mouse anti-human S100A8/S100A9 (27E10) mAb (Tebu-bio). After washing, cells were resuspended in PBS and analysis was performed on a FC500 instrument (Beckman Coulter).

### Production of S100A9 proteins

S100A9 monomers were purchased from Tebu-bio. S100A9 tetramer protein was produced by Protenia (Dr El Yahyaoui, Ifrane, Morocco) by standard procedures. Briefly, S100A9 (Calgranuline B) was cloned in pET3a vector and, after verification of the insert, BL21(DE3) Origami E. coli strain was transformed. Production of tetramer was tested after strain lysis and protein purification was verified by SDS-Page gel (Additional file 6: Figure S6). Proteins used in the experiments were LPS free.

### Analysis of intracellular cytokine production and CD107a expression by NK cells

After incubation with 1 μg/mL of either S100A9 monomer or tetramer for 4 hour, NK cells were overnight stimulated or not by HLA class-I-devoid K562 cells. CD107a assay was done as previously described [33]. After washing, cells were resuspended in PBS and analysis was performed on a LSRII instrument (BD Biosciences) on gated cells within the lymphocyte population. Within the CD3-CD56 + CD16+ population, we noted the independent expression of CD107a, TNF-α, IFN-γ for each sample following stimulation. We used PECy5-anti-CD107a (BD Biosciences), FITC-anti-IFN-γ (Beckman Coulter) and PE-anti-TNF-α (BD Biosciences) mAbs.

### Analysis of HIV-1 replication

Replication of HIV-1 was detected by measuring intracellular p24cbl by flow cytometry. After a formaldehyde/saponin-based fixation/permeabilization (IntraPrep, Beckman Coulter), HIV-1-infected MDDC or CD4+ T cells were stained with PE-anti-p24 (KC57 RD1) mAbs (Beckman Coulter) for 30 min. After washing, cells were resuspended in PBS until flow cytometric analysis was performed. Given that the CD4 antigen is down modulated after T cell activation and/or infection [34], we used the CD3 expression to distinguish CD3 + CD4+ T cells and CD3-CD56+ NK cells in co-culture. Infection of CD3+ T cells and MDDC with HIV-1 Bal strain was analysed in a LSRII instrument (Becton Dickinson). The presence of viral particles in the supernatant was determined by ELISA Test (RETROtek p24 Antigen ELISA kit, ZeptoMetrix Corp.) according to the manufacturers’ protocol.

### Inhibition of HIV-1 replication assay

Infections of MDDC and CD4+ T cells were performed with HIV-1 Bal strain at 10^-2^ moi. NK cells and infected target cells were co-cultured at a ratio E/T of 1:5 (20.10^3^NK/100.10^3^MDDC or 20.10^3^NK/100.10^3^CD4+ T cell per well, in a 96 well plate). CD4+ T cells were stimulated for 4 days with Phytohaemagglutinin-L (PHA-L) at 1 μg/ml in the presence of IL-2 at 100 U/ml prior the infection. HIV-1 infection of cells was recorded after 7 and 10 days of co-culture by measuring intracellular p24 by flow cytometry and assessing the amount of p24 antigen in supernant by ELISA test. When indicated, NK cells were stimulated or not for 1 h by full-length recombinant GST-tagged human S100A9 monomers (Tebu-bio) or by S100A9 tetramers (Protenia, SA) at 1 μg/ml.

### Role of the funding source

The French National Agency for AIDS and Hepatitis Research (ANRS) funded this study (ANRS 9070 study). The funder had no role in study design, data collection and analysis, decision to publish, or preparation of the manuscript.

### Statistical analyses

Data was analysed by non-parametric (Mann–Whitney test) and parametric tests (Student’s two-tailed paired t-test). The results are expressed as the sample mean ± SE.

## Competing interests

The authors declare that they have no competing interests.

## Authors’ contributions

Contributions: VA designed and performed research, analyzed data, and wrote the paper; JSC analyzed data and wrote the paper; UMN performed research, analyzed data, and wrote the paper; CD performed research; AG performed research and analyzed data; FBS designed research and analyzed data; DSA designed research, analyzed data, and wrote the paper. All authors read and approved the final manuscript.

## Supplementary Material

Additional file 1: Figure S1 S100A8 and S100A9 Gel Sequencing. **(A)** Eluted proteins from the column were separated by electrophoresis in non-reducing conditions and revealed by Coomassie blue staining. Lane 3, Seeblue® Plus2 Pre-Stained Standard, lanes 1–2, subsequent eluted fractions of HIV-1-infected MDDC lysates after pre-incubation with the CD85j-Fc-coupled gel. **(B)** Protein sequencing results are summarized: S100A8/S100A9 (Band 01), S100A9 (Bands 02a and 02b) and S100A8 (Band 03) proteins. Click here for file

Additional file 2: Figure S2 ELISA-based CD85j/S100A9 proteins binding assay. CD85j-Fc coated on the wells of a microtiter plate was incubated with increasing amounts of S100A9 monomer or tetramer proteins. Click here for file

Additional file 3: Figure S3 HIV-1 dose-dependent modulations of S100A8, S100A9 and S100A8/S100A9 at the surface of MDDC. Statistical analyses showing the spearman correlation between the frequency of p24+ MDDC and the surface expression of S100A8 **(A)**, S100A9 **(B)**, or S100A8/S100A9 complex **(C)**. MDDC were infected with different multiplicity of HIV-1 infection and stained after 7 days of culture. Each dot represents one flow cytometry analysis from a different individual. Click here for file

Additional file 4: Figure S4 Expression of S100A9 protein at the surface of MDDC in response to productive or non-productive HIV-1 infection. **(A)** Cumulative results showing S100A9 and expression at the surface of uninfected (Control Media), replicating (HIV-1 p24+) or not (HIV-1 p24-) HIV-1-infected MDDC, 7 days of culture. **(B)** Expression S100A9 on the surface of MDDC p24- in the context of a productive (HIV-1, HIV-1ΔNef, or HIV-1ΔVpu) or a non-productive HIV-1 infection (HIV-1ΔEnv VSV), compared to the condition of non-infection (Control Media). Results are expressed as mean ± SE of percentage of MDDC expressing S100A9 at the surface. Results of 6 independent experiments are summarized. * p < 0.05. Click here for file

Additional file 5: Figure S5 Quantification and analysis of the purification of S100A9 tetramers by 12% SDS-PAGE. Lane 1: 0.25 μg BSA; Lane 2: 0.5 μg BSA; Lane 3: 1 μg BSA; Lane 4: 2 μg BS; Lane 5: MM; Lane 6: 1 μL of eluted solution; Lane 7: 2 μL of eluted solution; Lane 8: 3 μL of eluted solution. Click here for file

Additional file 6: Figure S6 Increase in TNF α production. Click here for file
