# Supplementary figures and images for: S100A9 protein is a novel ligand for the CD85j receptor and its interaction is implicated in the control of HIV-1 replication by NK cells
Source: Retrovirology. 2013 Oct 24;10:122. doi: 10.1186/1742-4690-10-122 (PMC3826667; doi:10.1186/1742-4690-10-122)

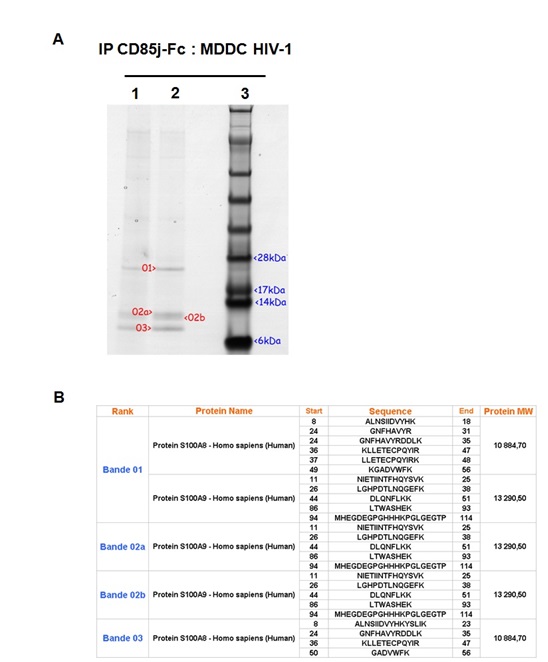

Supplement: Additional file 1: Figure S1 — S100A8 and S100A9 Gel Sequencing. (A) Eluted proteins from the column were separated by electrophoresis in non-reducing conditions and revealed by Coomassie blue staining. Lane 3, Seeblue® Plus2 Pre-Stained Standard, lanes 1–2, subsequent eluted fractions of HIV-1-infected MDDC lysates after pre-incubation with the CD85j-Fc-coupled gel. (B) Protein sequencing results are summarized: S100A8/S100A9 (Band 01), S100A9 (Bands 02a and 02b) and S100A8 (Band 03) proteins. [file 1742-4690-10-122-S1.jpeg]

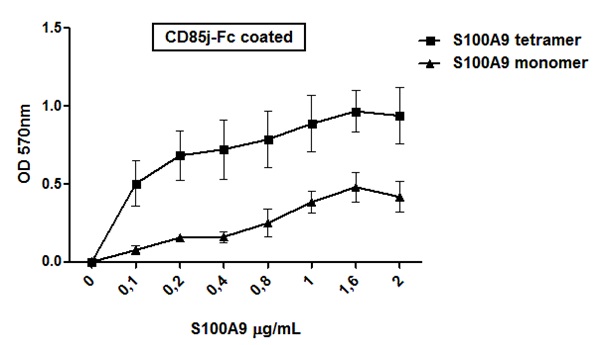

Supplement: Additional file 2: Figure S2 — ELISA-based CD85j/S100A9 proteins binding assay. CD85j-Fc coated on the wells of a microtiter plate was incubated with increasing amounts of S100A9 monomer or tetramer proteins. [file 1742-4690-10-122-S2.jpeg]

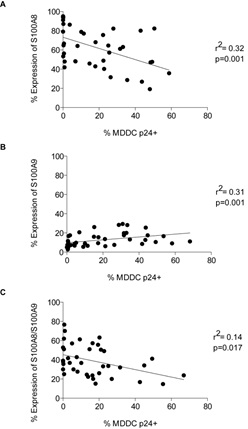

Supplement: Additional file 3: Figure S3 — HIV-1 dose-dependent modulations of S100A8, S100A9 and S100A8/S100A9 at the surface of MDDC. Statistical analyses showing the spearman correlation between the frequency of p24+ MDDC and the surface expression of S100A8 (A), S100A9 (B), or S100A8/S100A9 complex (C). MDDC were infected with different multiplicity of HIV-1 infection and stained after 7 days of culture. Each dot represents one flow cytometry analysis from a different individual. [file 1742-4690-10-122-S3.jpeg]

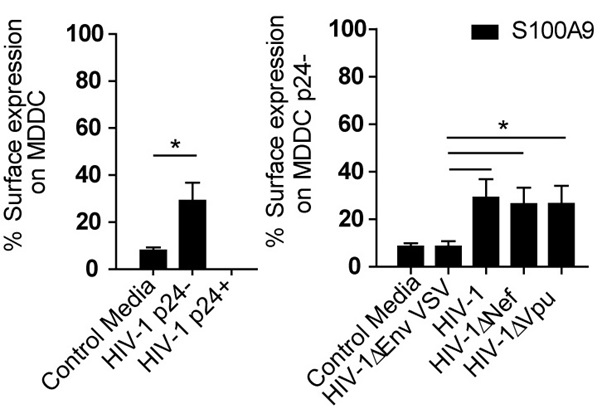

Supplement: Additional file 4: Figure S4 — Expression of S100A9 protein at the surface of MDDC in response to productive or non-productive HIV-1 infection. (A) Cumulative results showing S100A9 and expression at the surface of uninfected (Control Media), replicating (HIV-1 p24+) or not (HIV-1 p24-) HIV-1-infected MDDC, 7 days of culture. (B) Expression S100A9 on the surface of MDDC p24- in the context of a productive (HIV-1, HIV-1ΔNef, or HIV-1ΔVpu) or a non-productive HIV-1 infection (HIV-1ΔEnv VSV), compared to the condition of non-infection (Control Media). Results are expressed as mean ± SE of percentage of MDDC expressing S100A9 at the surface. Results of 6 independent experiments are summarized. * p < 0.05. [file 1742-4690-10-122-S4.jpeg]

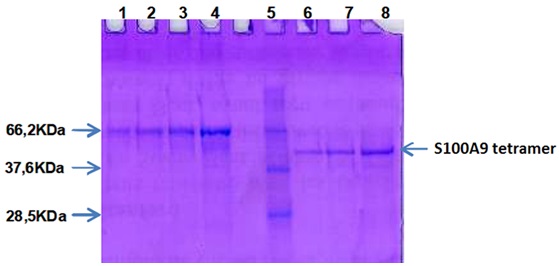

Supplement: Additional file 5: Figure S5 — Quantification and analysis of the purification of S100A9 tetramers by 12% SDS-PAGE. Lane 1: 0.25 μg BSA; Lane 2: 0.5 μg BSA; Lane 3: 1 μg BSA; Lane 4: 2 μg BS; Lane 5: MM; Lane 6: 1 μL of eluted solution; Lane 7: 2 μL of eluted solution; Lane 8: 3 μL of eluted solution. [file 1742-4690-10-122-S5.jpeg]

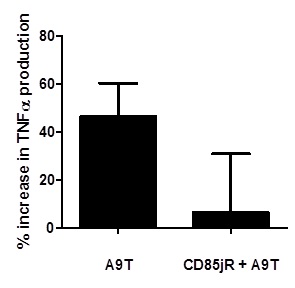

Supplement: Additional file 6: Figure S6 — Increase in TNF α production. [file 1742-4690-10-122-S6.jpeg]
